# Why bother with a COST Action? The benefits of networking in science

**DOI:** 10.1186/1753-4631-4-S1-S12

**Published:** 2010-06-03

**Authors:** Kalliopi Kostelidou, Fabio Babiloni

**Affiliations:** 1 COST Office, Brussels, 149 Ave. Louise B1050-Brussels, Belgium; 2 University of Rome Sapienza, Department of Physiology and Pharmacology, Rome, Italy

## Abstract

A COST Action is a consortium of -mainly- European scientists (but open to international cooperation) working on a common research area, with the same subject; COST provides funding to the Actions for networking and dissemination activities, thus the participating scientists must have secured research funding from other national or European sources. COST funding is in the scale of approximately 100 kEuros per year and in this vein, it is often criticized both in that it does not fund research and the core science and in that its funding is ‘limited’. However, COST with its instruments is an integral pillar of the European Research Area, and it is through its mission that a variety of aspects of the research environment, fundamental to the success of the research, are catered for; these include scientific networking, collaboration/exchange/training and dissemination activities. Through fast procedures, proposals are evaluated and approved for funding in less than one year from submission date and Actions become operational immediately, managed on flexible management. In this way, COST contributes to reducing the fragmentation in European research investments, while opening the European Research Area to cooperation worldwide. COST Actions have an excellent record of building the critical mass for follow up activities in the EU FP or other similarly competitive programmes.

## Background

Comparing the amounts (normally in the range of a few millions of Euros) on the calls of the FP7 research funding schemes (People, Cooperation, European Research Council-ERC) to the corresponding ones by COST (**C**ooperation in **S**cience and **T**echnology, http://www.cost.eu), an average of 100 kEuros per year, the spontaneous question pops to the mind of the reader: ‘why should I bother with COST?’.

This short article provides information on COST, its structure and governance, the application and selection procedures and some general information on what can be expected and achieved in the frame of a successful COST Action. The aim is to show that COST is a fast and flexible tool for enabling collaborative research across Europe and beyond which can be the basis for capacity building, strong, sustainable and successful consortia.

## About COST

COST is an intergovernmental framework for European **Co**operation in **S**cience and **T**echnology. COST allows the coordination of nationally-funded research on a European level and thus contributes to reducing the fragmentation in European research investments, while at the same time opening the European Research Area to cooperation worldwide. The mission of COST is to ensure that Europe holds a strong position in the field of scientific and technical research for peaceful purposes by increasing European cooperation and interaction in this field. This research initiative makes it possible for the various national facilities, institutes, universities and private industry to work jointly on a wide range of Research and Development (R&D) activities. Together with EUREKA and the EU FP programmes, COST is one of the three pillars of joint European research initiatives, with COST being the oldest one (established 1971).

The main COST instrument is the COST Action: this is a collaborative research network of scientists working on the same subject. Actions receive funding for a period of four years to develop cooperation by way of meetings, workshops, scientific exchanges, training schools, joint publications and dissemination activities.

COST has nine scientific and technical Domains which are clustered in three Clusters (Table 1). In addition, COST is unique in that it invites multi- and inter-disciplinary proposals under the ‘Trans-Domain’ area; proposals scanning across different areas and thus not fitting in a single Domain are submitted in the Trans-Domain track.

**Table 1:** COST scientific domains

Cluster of Life Sciences	BMBS - Biomedicine and Molecular Biosciences FA - Food and Agriculture FPS - Forests, their Products and Services
Cluster of Natural Sciences	CMST - Chemistry and Molecular Sciences and Technologies ESSEM - Earth System Science and Environmental Management MPNS - Materials, Physical and Nanosciences

Cluster of Science and Society	ISCH - Individuals, Societies, Cultures and Health ICT - Information and Communication Technologies TUD - Transport and Urban Development

Trans Domain area (not a separate Domain currently) – allows for multidisciplinary proposals which cannot be submitted to a single Domain.	

## COST structure

COST is governed by the COST Member States and its organisation reflects its intergovernmental nature.

COST Ministerial Conferences: The key decisions are taken at COST Ministerial Conferences, which are held on average every five years.

Committee of Senior Officials (CSO): The Committee of Senior Officials (CSO) is the main decision-making body responsible for the strategic development of COST, but also decides on the proposed new Actions following the Open Call. The CSO is constituted by two representatives per COST Member State. One of the two representatives to the CSO is usually the COST National Coordinator (CNC).

COST National Coordinator (CNC): The role of the COST National Coordinator (CNC) is to nominate delegates to the COST Domain Committees (DC) and the Management Committees (MC) of the COST Actions. The CNC additionally provides advice on all COST related matters (e.g. Actions, participation, Open Call), provides the liaison between the scientists and institutions in his/her country.

Executive Group of the CSO (JAF): The Executive Group of the CSO is referred to as JAF, and prepares the CSO meetings and is responsible for some every day decisions delegated by the CSO. The group consists of the President and the Vice-President of the CSO as well as five other delegates from the CSO who are chosen to represent different COST countries for a maximum duration of three years.

Domain Committee (DC): the Domain Committees (DC) consist of experts in the respective research domain and are nominated by the CNC. The key DC role is the quality control of the Domain Actions (assessment, monitoring, evaluation). Additionally, the DC is supervising the strategic development of their respective Domain.

In addition to the DC members, each country nominates a small number of experts who form a pool of expertise to be drawn upon for assessments.

Management Committee (MC): The Management Committee (MC) is the decision-making body for each Action; the MC is formed of by national experts who participate in an Action. The experts are nominated by their countries’ CNC members. The role of the MC is to decide upon and coordinate the activities of the Action.

COST Secretariat: The General Secretariat of the Council of the European Union provides the secretariat for the CSO and the JAF.

COST Office: The COST Office in Brussels is provided by the European Science Foundation (ESF, which acts as the implementing agent for COST). It supports the scientific activities, e.g. the DCs and Actions’ activities, and implements the decisions taken by the CSO.

## COST characteristics

The following are key COST characteristics:

1. a ‘bottom-up’ approach- the idea and subject of a COST Action comes from the European scientists themselves and there are no predetermined thematic priorities in the COST Open Calls

2. participation is “à la carte” – only the parties (=countries or other bodies) which are interested to an Action nominate scientists to take part in an Action based on scientific merit;

3. equality of access (participation is open to all COST countries) and scientists can join after the Action has been approved and funded

4. a flexible structure (easy implementation and lean management of the research initiatives); a web-based tool (ecost) has been developed to be user-friendly and easily managed to provide Actions the ability to administer and report on their activities.

## How does COST work? The procedure to get funding

COST launched an Open Call for proposals in 2006, following a two-stage procedure. As of 2007, there are two collection dates per year, in March and September. All the information required for the submission can be found in the key document COST doc. 205/08 ‘Guidelines for the Assessment, Monitoring, Evaluation and Dissemination of Results of a COST Action’ (to be accessed under the http://www.cost.eu/participate link). The criteria for the evaluation of the proposals are also to be found there.

There are no thematic priorities and scientists can submit their proposal on any subject. Proposals are submitted per Domain (the proposer indicates the relevant Domain), but the COST office reserves the right to allocate the proposal to a different Domain as appropriate.

A pre-proposal of 1500 words, is evaluated by the Domain Committees (based on defined and published criteria) and the top pre-proposals are invited for a full technical annex submission. This second-stage proposal is longer, more detailed and also includes information on the interested future participants for the Action, if it is to be approved. These second-stage proposals are evaluated by an external panel of experts, including specialists in the fields of the proposals under evaluation. Proposals approved by the panel are then invited for oral presentation to the Domain Committees. It is here that the final ranking per Domain is made for the proposals. The final stage involves a consensus meeting between all three Chairs of the Domain Committees of each Cluster and the Chair of the Trans-Domain to agree on a consensus final ranking of Actions per Cluster to be recommended for approval (including the relevant proposals from the Trans-Domain which are allocated to one Domain for administrative purposes).

The successful proposals are approved for funding by the CSO and enter into force when at least five COST parties (countries) indicate their interest to accept the Memorandum of Understanding of the Action. When at least five parties have accepted the MoU, a kick-off meeting can be convened by the COST Office and this signals the start date of the Action, which can then run normally for four years. 

Funding to each Action takes into consideration the number of participating parties and normally averages at 100 k Euros per year. Funding cannot be provided for any kind of research activity, including salaries, consumables etc. COST supports all kinds of networking/collaboration within the Action and more specifically meetings (either organised by the Action or joined to other events), workshops, conferences, exchange visits of short periods (normally up to three months), training schools (either hands-on or lecture-based ones), publications and can also provide partial support for a web site or a database.

Across the 7 Collection dates until early 2010 and across all COST Domains, the success rate for the first stage pre-proposals (i.e. the chance to be invited for a full proposal submission) is approximately 18%, while for the second stage proposals, (i.e. the chance to be approved for funding after invited for full proposal submission), this is around 40% (even though there are variations among the Domains). The filter in the first stage needs to be a harsh one, as the available budget would not allow otherwise; as a norm, approximately 2 times more proposals are invited for full stage submission.

## Discussion

Since its establishment 40 years ago, COST has developed into one of the largest frameworks for research co-operation in Europe and is a valuable mechanism co-ordinating research activities in Europe.

**Figure 1:**
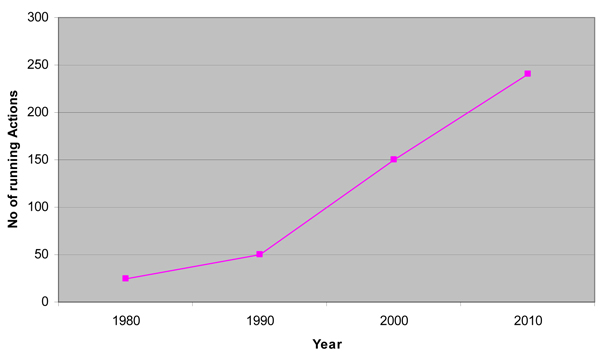
**Figure shows the number of the running Actions in the different COST domains from 1980 to 2010.**

Today (2010) COST has around 240 running Actions (running at any time of the year), a number which represents a nearly 400% increase from the 80s (see Figure 1 for an evolution of COST running Actions) COST involves approx. 32,000 scientists from 36 European member countries, more than 250 participating institutions from 35 non-member countries and Non Governmental Organisations and has reciprocal agreements with Australia, New Zealand and South Africa. COST has additionally developed a dedicated Trans-Domain channel for inviting and evaluating multi-disciplinary proposals. 

As a precursor of advanced multidisciplinary research, COST plays a vital role in building a European Research Area. It anticipates and complements the activities of the EU Framework Programmes, constituting a “bridge” towards the scientific communities of emerging countries. Due to its flexible structures, it allows the fast approval of new Actions and a range of their activities following their funding.

Some facts and figures: in 2008, COST supported a total of 859 scientific workshops and meetings, which equals an average of 4.1 per working day. More than 31000 participants attended these meetings. Additionally, COST funded 785 scientific exchange visits, corresponding to an average of 3.7 exchanges starting per working day of the year. COST supported a total of 38 training schools with 800 funded participants and 276 high ranked publications (this number corresponds only to the publications funded by COST and does not include the joint publications where COST was acknowledged, which are in the range of thousands.)

### Why to bother with a COST application

So, why bother with a COST Action application?

We would like to simply present some facts below which can illustrate the merit for the COST funding scheme, from a point of view of a scientist.. A standard approved proposal in EU Framework Programme (as an example a STREP proposal) returns a substantial amount of money for each participant when compared to the funding of a COST Action and importantly, research funds. For instance, each participant of a STREP proposal receives usually an amount of 400-500Keuros on a total of three years. It is indeed true that the individual annual income for a partner of a EU STREP project is about equal to the funding of an COST Action with 20 countries participants, for four years.

However, in the STREP proposal the money for funding networking with the other participants (on average 4-6 participants per STREP consortium) are limited to about 30-50K for the three years of the project. During the lifetime of a COST Action, a total of 8-10 large workshops with 60-70 scientists per workshop can be supported. This means an occasion to meet and discuss specific themes of science related to the Action with a total of about 600 scientists in 4 years. The continuity of these meetings along four years supports in depth discussion of scientific matters, creates new cooperations (especially with scientists who join the Action after its start) and sustains pre-existing collaborations.

So, while undoubtedly the EU funding schemes are of paramount importance, COST with its ‘lower’ budget serves a critical role in the ERA. The essence of a COST Action is the generation of a durable network of scientists that meet one to each other regularly. A regular sequence of scientific meetings means a regular exchange of scientific ideas, that are the most valuable currency provided to each scientist. Is that particular currency (i.e the scientific ideas exchanged along the years with regular meeting with hundred of colleagues) that provide an answer to the initial question of this paper.

## Competing interests

There are no competing interests (financial, political, personal, religious, ideological, academic, intellectual, commercial or any other) to declare in relation to this manuscript.

